# Prognostic role of matrix metalloproteinases in bladder carcinoma: a systematic review and meta-analysis

**DOI:** 10.18632/oncotarget.15907

**Published:** 2017-03-04

**Authors:** Chenkui Miao, Chao Liang, Jundong Zhu, Aiming Xu, Kai Zhao, Yibo Hua, Jianzhong Zhang, Wei Chen, Chuanjian Suo, Chao Zhang, Yiyang Liu, Shifeng Su, Zengjun Wang

**Affiliations:** ^1^ State Key Laboratory of Reproductive Medicine and Department of Urology, The First Affiliated Hospital of Nanjing Medical University, Nanjing, People’s Republic of China; ^2^ Department of Urology, Nanjing First Hospital, Nanjing Medical University, Nanjing, People’s Republic of China

**Keywords:** matrix metalloproteinases, bladder cancer, prognosis, meta-analysis

## Abstract

Recent studies have shown that matrix metalloproteinases (MMPs) might be a biomarker for predicting outcomes of bladder cancer. However, the prognostic value of overexpression of MMPs in bladder cancer is debatable and the studies are inconsistent. Therefore, this meta-analysis was performed to clarify the specific association and prognostic value of overexpression of MMPs in bladder carcinoma. Relevant studies were identified by searching PubMed, EMBASE, and the Web of Science. Pooled hazard ratios (HRs) with 95% confidence intervals (CIs) for disease-specific survival (DSS), overall survival (OS), disease/recurrence-free survival (DFS/RFS), and progression/metastasis-free survival (PFS/MFS) were analyzed to determine the prognostic value of MMPs. In total, eighteen applicable studies were included in this meta-analysis. We found that high expression of MMPs significantly correlated with a poor DSS and OS (HR=1.66; 95% CI = 1.38–2.01 and HR= 1.67; 95%CI= 1.26–2.22). MMPs also predicted tumor progression and metastasis with a pooled HR of 3.03 (95% CI 1.98–4.64). However, high MMPs expression had no pivotal impact on DFS/RFS (HR= 1.21; 95% CI= 0.96–1.53). With the purpose of better understanding the prognostic role of MMPs in patients wirh bladder carcinoma, we carried out this systematic review and meta-analysis.

## INTRODUCTION

Despite a declining occurrence rate in recent years, bladder cancer is still the most frequent malignancy of the urinary system worldwide. Its incidence had risen to be the ninth most common malignant tumor in 2008 [1]. Approximately 386, 000 new cases of bladder carcinoma and 150, 000 disease-specific deaths occur worldwide every year [2]. Recent studies have shown that around 30% of bladder cancer cases are muscle invasive bladder cancers (MIBC) while the other approximately 70% are non-muscle invasive bladder cancers (NMIBC) [3, 4].

Although some progress has been made in therapeutic approaches for bladder cancer, patients still experienced poor survival outcome, with high recurrence and mortality. The pathogenesis and progression of bladder cancer is complicated, and its occurrence and development seem to be influenced by many factors, such as multiple genes and external environmental factors [5, 6]. To improve the quality of patients’ individual care, it is essential to investigate prognostic factors for survival and recurrence of bladder cancer and identify novel techniques for diagnosis and treatment [7].

Matrix metalloproteinases (MMPs) family belongs to more than 25 zinc-dependent endogenous proteolytic enzymes and they have significant influences on tumor invasion and migration, such as extracellular matrix (ECM) degradation, loss of cellular adhesion, tumor angiogenesis, epithelial-to-mesenchymal transitions, and cellular proliferation [8, 9]. Recently, increasing evidence has verified that the activity of MMPs plays pivotal role in several physiological and pathological processes, such as the development of multiple carcinomas and angiocardiopathy [10–13]. There are numerous subtypes of MMPs, such as MMP1, MMP2, MMP3, MMP7, MMP9, and MMP14. Many of studies have evaluated levels of MMPs extensively in cancer patients, and reported vital roles of some MMPs as diagnostic and prognostic biomarkers in tumorigenesis. Previous systematic reviews have demonstrated that certain MMPs subtypes are associated with a poor outcome in stomach, breast, and ovarian cancer [14, 15]. Additionally, a number of basic and clinical studies have shown an relationship between MMPs and shortened survival in bladder cancer patients [16].

However, a few individual studies have proposed contradictory conclusions. For instance, Vasala et al. reported that increased expression of MMP9 correlated with longer overall survival and decreased recurrence rates of urinary bladder cancer. MMP9 may therefore serve as a favorable biomarker and its overexpression may predict better outcomes in bladder cancer patients [17]. The results was still controversial due to several limitations: small sample sizes, lower statistical veracity, and genuine heterogeneity. Therefore, we carried out a systematic review and meta-analysis to clarify the relevance of abnormal levels of MMPs on outcomes in patients with bladder carcinoma.

## RESULTS

### Summary of enrolled studies

**Figure 1:**
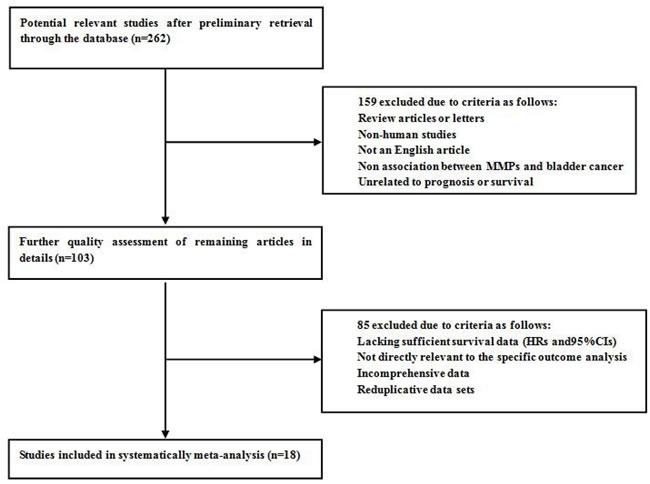
Flow diagram of literature search and selection process

As is shown in Figure 1, a total of 262 studies from PubMed, EMBASE, and the Web of Science were found to focus on the relationship between MMPs expression and bladder carcinoma. After initial screening of titles and abstracts, 159 studies were excluded because: they did not focus on the specific association of MMPs expression and bladder cancer, were review articles or non-English articles, or did not include human subjects. After applying this criteria to filter the remaining 103 studies, 85 potentially suitable studies were excluded because they lacked sufficient survival data (HRs and 95% CIs), were not relevant to the outcome analysis, did not report comprehensive data, or had reduplicative data sets. Ultimately, eighteen studies were considered applicable to this meta-analysis. The inclusion and exclusion reasons for the screened studies are summarized in detail in Figure 1.

Dominant features and results of the appropriate articles are listed in Table 1 and Table 2. To sum up, all studies included were published from 1996 to 2016. These studies investigated the association between bladder cancer and multiple MMPs types. Among these, there are seven studies focusing on MMP2, six studies focusing on MMP9, four articles concentrating upon MMP7, and three articles on MMP3. Furthermore, there exist two articles regarding MMP1, and one article each on MMP8, MMP11, and MMP14. Eleven of the studies focused on Caucasian populations, which mainly came from European countries, and seven focused on Asian populations, of which six from Japan primarily and one from China.

**Table 1:** Main characteristics of studies included in the meta-analysis

First author, publication year	MMP types	Case nationality	Dominant ethnicity	Median or mean age	Study design	Detected sample	Survival analysis	Source of HR	Maximum months of follow-up
Li, 2016	MMP11	China	Asian	65.8	R	tissue	DSS/MFS	Reported	175.8
Minami, 2014	MMP2	Japan	Asian	71	R	serum	DSS/RFS	Reported	166.4
Demery, 2014	MMP7	France	Caucasian	69	R	serum	OS	Reported	194.4
Olsson, 2012	MMP2	Sweden	Caucasian	73	R	tissue	RFS/PFS	Reported	192
Olsson, 2012	MMP9	Sweden	Caucasian	73	R	tissue	RFS	Reported	192
Szarvas, 2011	MMP7	Germany	Caucasian	65	R	serum	OS/DSS/MFS	Reported	148
Svatek, 2010	MMP1	America	Caucasian	64	R	serum	DSS	Reported	50
Svatek, 2010	MMP2	America	Caucasian	64	R	serum	DSS	Reported	50
Svatek, 2010	MMP3	America	Caucasian	64	R	serum	DSS	Reported	50
Svatek, 2010	MMP7	America	Caucasian	64	R	serum	DSS	Reported	50
Svatek, 2010	MMP8	America	Caucasian	64	R	serum	DSS	Reported	50
Svatek, 2010	MMP9	America	Caucasian	64	R	serum	DSS	Reported	50
Szarvas, 2010	MMP7	Germany	Caucasian	65	R	serum	OS/DSS/MFS	Reported	196
Sagara, 2010	MMP14	Japan	Asian	71	R	tissue	DSS/MFS	SC	195
Vasala, 2003	MMP9	Finland	Caucasian	67	R	tissue	OS/DSS/RFS	SC	120
Slaton, 2004	MMP9	America	Caucasian	65.5	R	tissue	DSS	Reported	168
Vasala, 2003	MMP2	Finland	Caucasian	66	R	tissue	OS/DSS/RFS	SC	120
Durkan, 2003	MMP9	England	Caucasian	70	R	tissue	DSS/PFS	Reported	39
Durkan, 2001	MMP1	England	Caucasian	70	R	urine	DSS/PFS	SC	39
Nakopoulou, 2001	MMP3	Greece	Caucasian	70	R	tissue	OS	SC	65
Hara, 2001	MMP9	Japan	Asian	65	R	tissue	RFS	SC	39
Kanayama, 1998	MMP2	Japan	Asian	69.7	R	tissue	DSS	SC	85.8
Gohji, 1998	MMP2	Japan	Asian	59	R	serum	DSS	SC	60
Gohji, 1996	MMP3	Japan	Asian	57	R	serum	DSS	SC	100

Study design is described as retrospective (R); MMPs, matrix metalloproteinases.

OS, overall survival; DSS, disease-specific survival; DFS, disease-free survival; RFS, recurrence-free survival; PFS, progression-free survival; MFS, metastasis-free survival.

SC, survival curve.

**Table 2:** HRs and 95% CIs of patient survival or cancer progression relating to MMPs expression in eligible studies

First author, publication year	MMP types	Main assay method	Cut-off value	Case number		OS		DSS/CSS		DFS/RFS		PFS/MFS	
				High expression	Low expression	HR(95%CI)	*P* value	HR(95%CI)	*P* value	HR(95%CI)	*P* value	HR(95%CI)	*P* value
Li, 2016	MMP11	IHC	median	170	170	NM	NM	3.027(1.406-6.516)	0.005	NM	NM	2.261(1.149-4.45)	0.018
Minami, 2014	MMP2	ElISA	mean	48	47	NM	NM	2.62(1.04-6.58)	0.04	0.77 (0.33-1.81)	>0.05	NM	NM
Demery, 2014	MMP7	ElISA	mean	16	39	2.1(1.1-4.4)	0.035	NM	NM	NM	NM	NM	NM
Olsson, 2012	MMP2	IHC	NM	18	185	NM	NM	NM	NM	1.27 (0.83-1.92)	0.27	NM	NM
Olsson, 2012	MMP9	IHC	NM	38	163	NM	NM	NM	NM	1.56 (1.01-2.38)	0.046	NM	NM
Szarvas, 2011	MMP7	ElISA	mean	46	32	2.264 (1.235–4.148)	0.008	1.906 (1.006–3.614)	0.048	NM	NM	2.037(0.625-6.636)	0.238
Svatek, 2010	MMP1	ElISA	median	NM	NM	NM	NM	1.10(0.54-2.22)	0.801	NM	NM	NM	NM
Svatek, 2010	MMP2	ElISA	median	NM	NM	NM	NM	0.66(0.32-1.38)	0.272	NM	NM	NM	NM
Svatek, 2010	MMP3	ElISA	median	NM	NM	NM	NM	0.97(0.50-1.86)	0.916	NM	NM	NM	NM
Svatek, 2010	MMP7	ElISA	median	NM	NM	NM	NM	2.24(1.12-4.47)	0.022	NM	NM	NM	NM
Svatek, 2010	MMP8	ElISA	median	NM	NM	NM	NM	1.24(0.54-2.85)	0.605	NM	NM	NM	NM
Svatek, 2010	MMP9	ElISA	median	NM	NM	NM	NM	1.08(0.55-2.14)	0.82	NM	NM	NM	NM
Szarvas, 2010	MMP7	ElISA	median	29	50	2.087(1.201–3.627)	0.009	2.351(1.251–4.418)	0.008	NM	NM	3.381(1.370–8.347)	0.008
Sagara, 2010	MMP14	IHC	median	43	42	NM	NM	1.84(0.40-8.53)	0.132	NM	NM	NM	NM
Vasala, 2003	MMP9	IHC	median	33	54	0.61 (0.28-1.30)	0.132	1.12(0.32-3.96)	0.272	0.64 (0.37.-1.11)	0.079	NM	NM
Slaton, 2004	MMP9	ISH	median	17	47	NM	NM	1.76(1.03, 3.02)	0.04	NM	NM	NM	NM
Vasala, 2003	MMP2	IHC	median	35	19	1.24(0.46-3.37)	0.09	1.19(0.27-5.15)	0.04	1.14(0.15-8.66)	0.07	NM	NM
Durkan, 2003	MMP9	IHC	median	64	42	NM	NM	7.56(1.22–12.36)	0.022	NM	NM	6.22 (1.51–10.37)	0.005
Durkan, 2001	MMP1	ElISA	median	21	110	NM	NM	5.53(1.07-28.58)	0.02	NM	NM	3.23(0.56-18.69)	0.04
Nakopoulou, 2001	MMP3	IHC	median	14	45	1.66(0.75-3.71)	0.088	NM	NM	NM	NM	NM	NM
Hara, 2001	MMP9	Northen Blot	median	16	35	NM	NM	NM	NM	2.01(0.95-3.66)	0.041	NM	NM
Kanayama, 1998	MMP2	qRT-PCR	mean	21	20	NM	NM	6.16(1.44-28.86)	<0.001	NM	NM	NM	NM
Gohji, 1998	MMP2	EIA	mean	22	31	NM	NM	1.50(0.41-5.40)	0.2224	NM	NM	NM	NM
Gohji, 1996	MMP3	EIA	mean	23	30	NM	NM	1.43(0.49-4.14)	<0.02	NM	NM	NM	NM

HR and 95% CI calculated from survival curves or article reports.

HR, hazard ratio; CI, confidence interval; MMPs, matrix metalloproteinases; SC, survival curve; NM, not mentioned.

OS, overall survival; DSS, disease-specific survival; DFS, disease-free survival; RFS, recurrence-free survival; PFS, progression-free survival; MFS, metastasis-free survival.

IHC, immunohistochemistry; ELISA, enzyme linked immunoassay; RT-qPCR, reverse transcriptase-quantitative PCR; EIA, enzyme immunoassay; ISH, in situ hybridization.

Six studies reported patient overall survival (OS), fourteen focused on disease-specific survival/cancer specific survival (DSS/CSS), five studies covered disease/recurrence-free survival (DFS/RFS), while another five articles investigated progression/metastasis-free survival (PFS/MFS). In addition, all of included studies were retrospective in design, and the maximal follow-up period ranged from 39 to 192 months.

Tissue samples were used to determine MMPs expression in ten studies, serum samples were used in seven studies, and urine samples were used in one study. Enzyme linked immunoassay (ELISA) and immunohistochemistry (IHC) were used in the majority of all eligible studies to detect MMPs expression. A few studies used northern blots, reverse transcriptase-quantitative polymerase chain reaction (RT-qPCR), or an enzyme immunoassay (EIA). Although a few articles did not mention the cut-off point defining MMPs expression, median/mean value served as the dividing grade in most included studies.

### OS associated with MMPs expression

**Figure 2:**
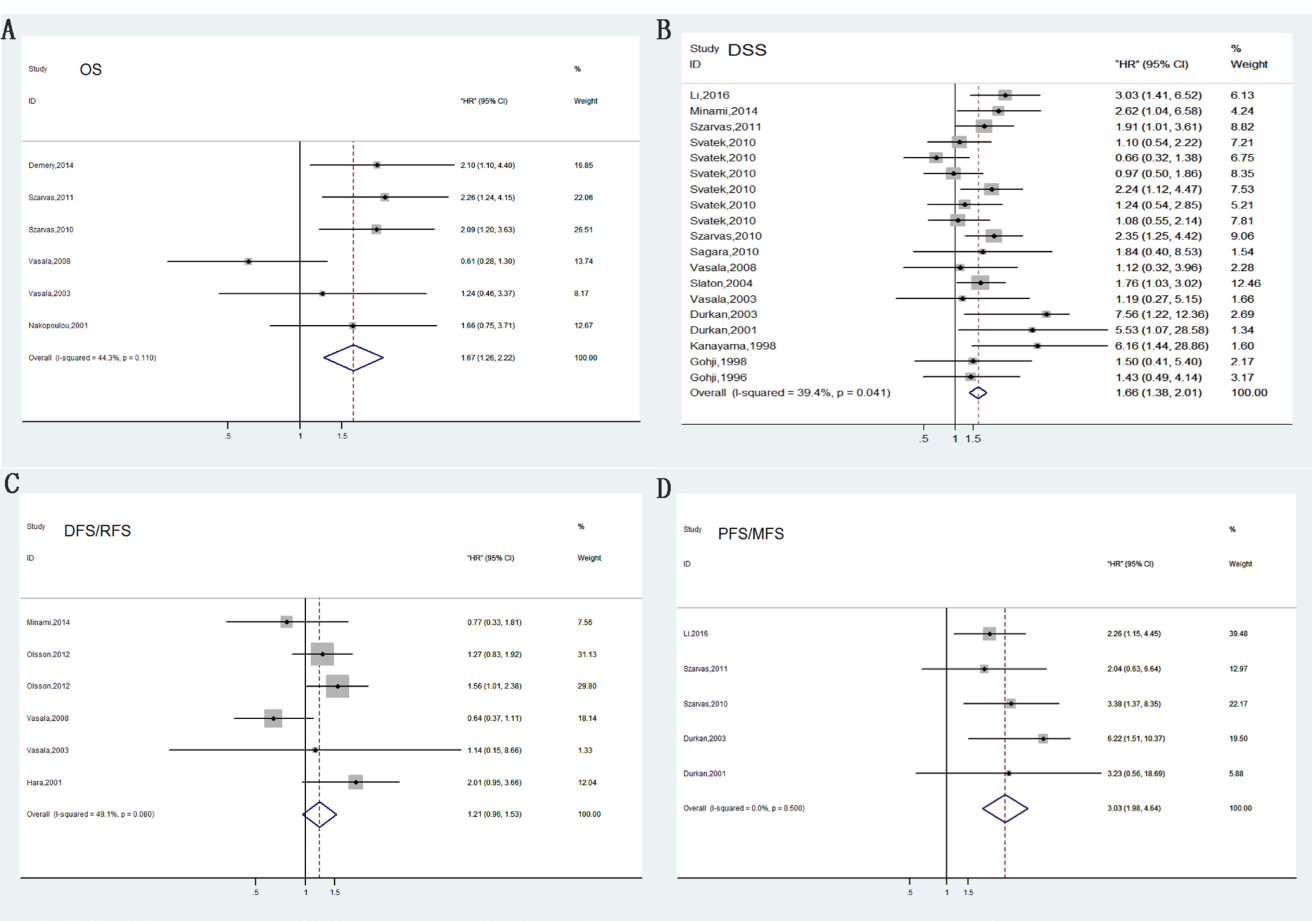
Forrest plots of merged analyses of high MMP expression as compared to low expression Survival data are reported as overall survival (OS) **A**. and disease-specific survival (DSS) or cancer-specific survival (CSS) **B**., disease-free survival (DFS) or relapse-free survival (RFS) **C**., progress-free survival (PFS) or metastasis-free survival (MFS) **D**.

Among the seven studies reporting OS, a fixed-effects model was used to analyze the data because of no significant heterogeneity (*P* = 0.110, *I*2 = 44.3%). Therefore, we concluded that MMPs overexpression was significantly associated with poor OS outcome in bladder carcinoma(HR= 1.67; 95% CI= 1.26–2.22; Figure 2A).

**Figure 3:**
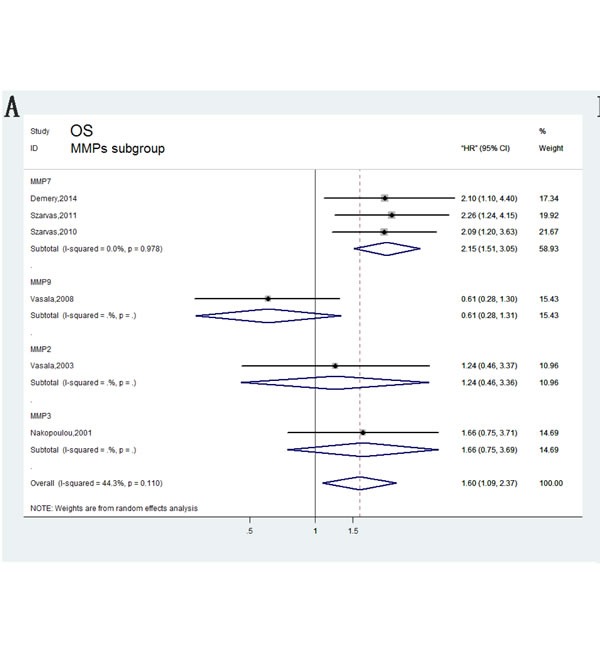

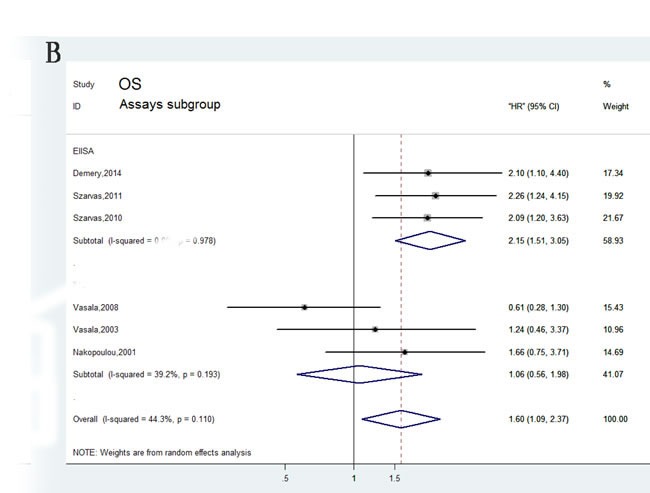

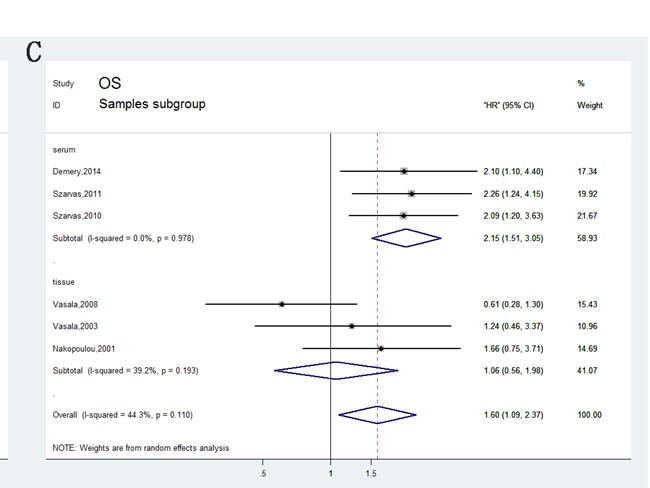
Forest plots of subgroup analysis of the OS **A**. stratified by MMPs subgroups; **B**. stratified by Assay methods; **C**. stratified by Sample subgroups.

Furthermore, subgroup analyses were used to determine the effect of MMPs up-regulation on bladder carcinoma. A pooled analysis of three studies in the MMP7 subgroup indicated that increased MMP7 expression was significantly correlated with reduced OS (Figure 3A). However, the correlation between other MMPs and overall survival in bladder cancer patients was ambiguous due to insufficient studies. Upon samples type, using serum was significantly associated with overall death rates with a pooled HR of 2.15 (95%CI= 1.51–3.05), whereas tissue showed no statistical significance (HR= 1.04, 95% CI = 0.64–1.69; Figure 3C).

### DSS associated with MMPs expression

**Figure 4:**
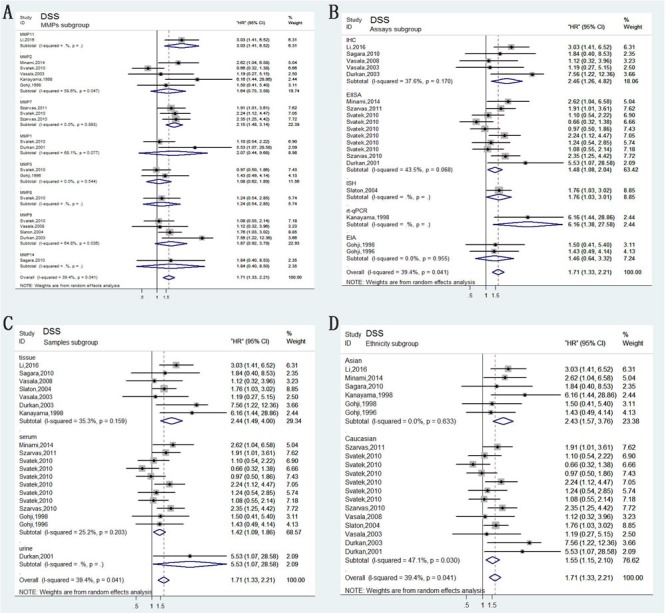
Forest plots of subgroup analysis of the DSS/CSS **A**. stratified by MMPs subgroups; **B**. stratified by Assay methods; **C**. stratified by Sample subgroups; **D**. stratified by Ethnicity subgroups.

Compared with OS, DSS can better reflect the outcomes of bladder carcinoma. Fourteen of the studies analyzed DSS. No heterogeneity between these studies was observed (*P* = 0.055, *I*2 = 35%); thus, a fixed-effects model was applied to calculate a pooled HR along with 95% CI. Our analysis revealed that high expression of MMPs correlated with shorter DSS (HR=1.66, 95%CI=1.38–2.01; Figure 2B). In addition, characteristics like MMPs type, assay methods, dominant ethnicity, and type of samples were divided into subgroups for analysis; calculated results were exhibited in detail in Figure 4.

### DFS/RFS associated with MMPs expression

**Figure 5:**
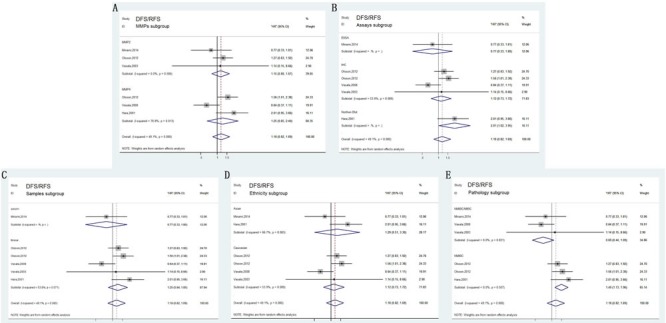
Forest plots of subgroup analysis of the DFS/RFS **A**. stratified by MMPs subgroups; **B**. stratified by Assay methods; **C**. stratified by Sample subgroups; **D**. stratified by Ethnicity subgroups; **E**. stratified by Pathology subgroups.

In total, six studies included in DFS/RFS analysis indicated no valuable role of increased MMPs expression for predicting DFS/RFS (HR= 1.21, 95% CI= 0.96–1.53, Figure 2C), which was determined by a fixed-effects model (*P* = 0.080, *I*2 = 49.1%). In anglysis of pathology subgroup, Olsson in 2012 and Hara in 2001 focusing on non muscle-invasive bladder cancer (NMIBC) revealed that MMPs predicted high risk of recurrence (HR= 1.49, 95%CI= 1.13-1.96, Figure 5E). However, other three studies did not differentiate muscle-invasive disease (MIBC) and non muscle-invasive (NMIBC) and obtained insignificant results. Furthermore, results of other subgroup analysis were also depicted in Figure 5.

### Cancer progression associated with MMPs expression

**Figure 6:**
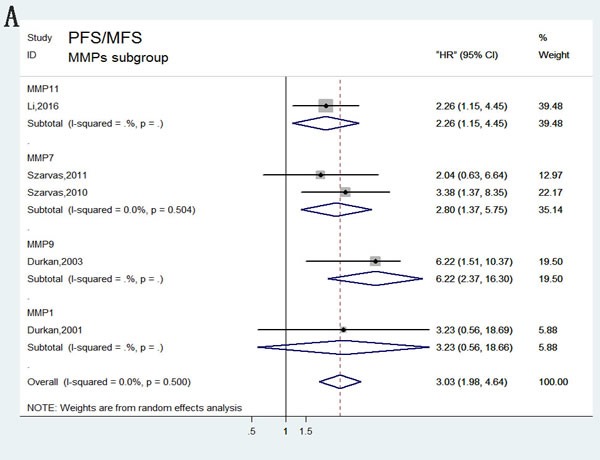

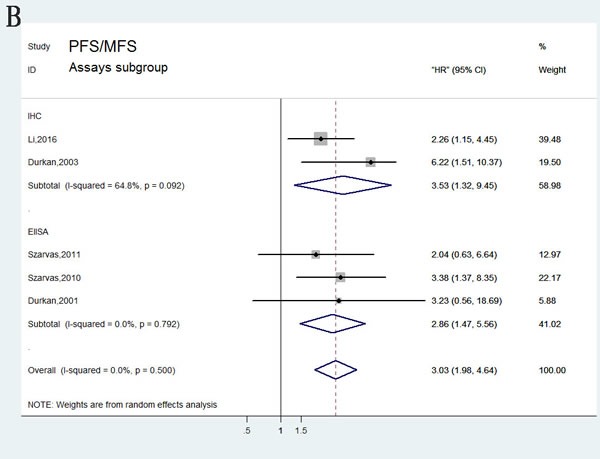

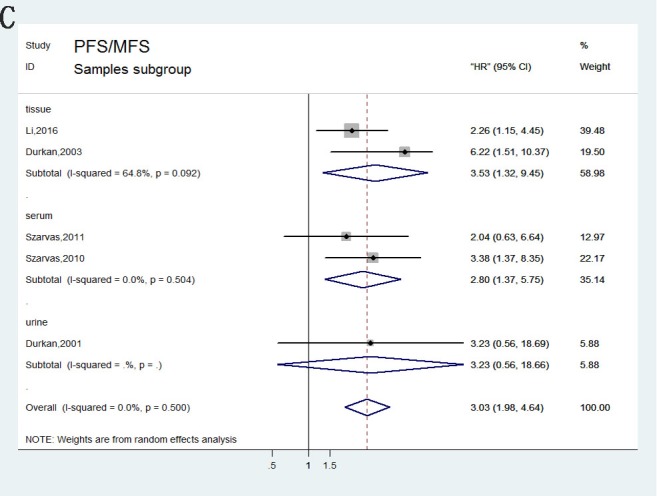
Forest plots of subgroup analysis of the PFS/MFS **A**. stratified by MMPs subgroups; **B**. stratified by Assay methods; **C**. stratified by Sample subgroups.

Generally, bladder cancer progression was evaluated by combining tumor recurrence and metastasis. In total, five articles investigating four different MMPs types (MMP1, MMP7, MMP9, and MMP11) reported a relationship between abnormal levels of MMPs and bladder tumor progression. A pooled HR (3.03) and 95% CI (1.98–4.64) were obtained with no heterogeneity (Figure 2D). Our meta-analysis revealed the association between abnormal MMPs expression and cancer progression. Stratified analyses were performed for MMPs, sample types, and assay methods, and similar results were obtained (Figure 6).

### Sensitivity analyses

**Figure 7:**
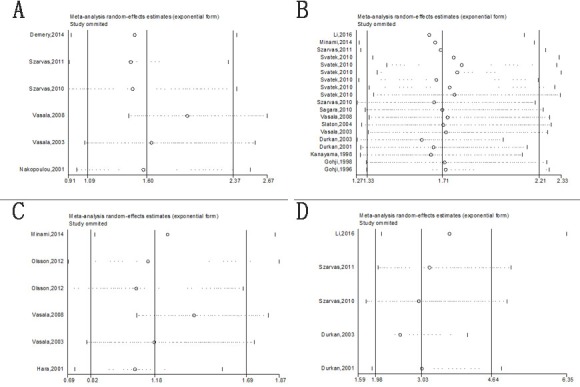
Sensitivity analysis under the specific model **A**. effect of individual studies on the pooled HR for OS; **B**. effect of individual studies on the pooled HR for DSS/CSS; **C**. effect of individual studies on the pooled HR for DFS/RFS. **D**. effect of individual studies on the pooled HR for PFS/MFS.

In order to reduce the effect of individual studies on final conclusions and evaluate the stability of results, a sensitivity analysis was performed by fixed-effect model. This test indicated that for DSS, DFS/RFS, and PFS/MFS, our results did not tend to exhibit alterations when an individual study was excluded (Figure 7). However, our analysis discovered that Vasala's (2008) investigation had an obvious influence on OS result [17]. After excluding this data, a more convincing pooled HR and 95% CI was obtained (HR= 1.96, 95% CI= 1.45–2.67, Figure 9A) and heterogeneity decreased significantly (*P* = 0.864, *I*2 = 0%). In addition, results of sensitivity analyses and publication bias no longer changed anymore (Figure 9B, 9C).

**Figure 8:**
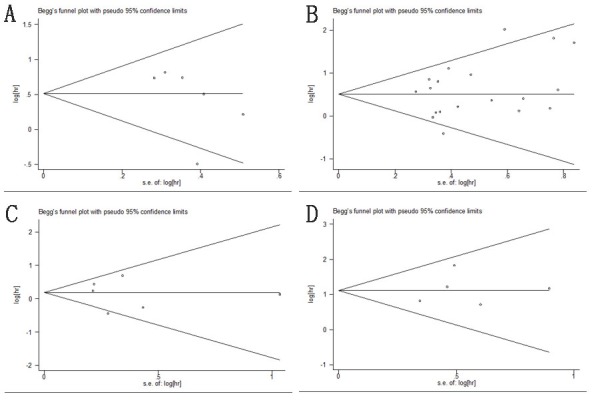
Begg's funnel plots of publication bias test **A**. OS; **B**. DSS/CSS; **C**. DFS/RFS; **D**. PFS/MFS.

**Figure 9:**
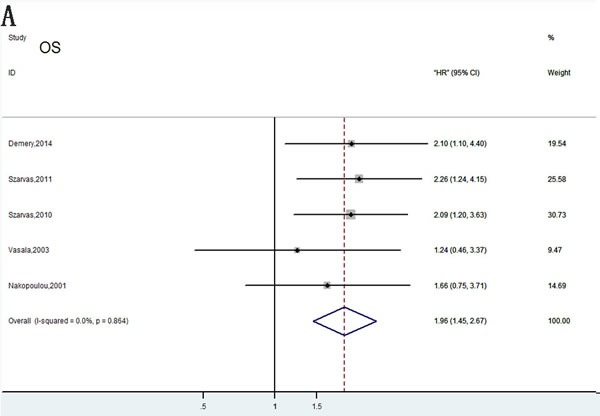

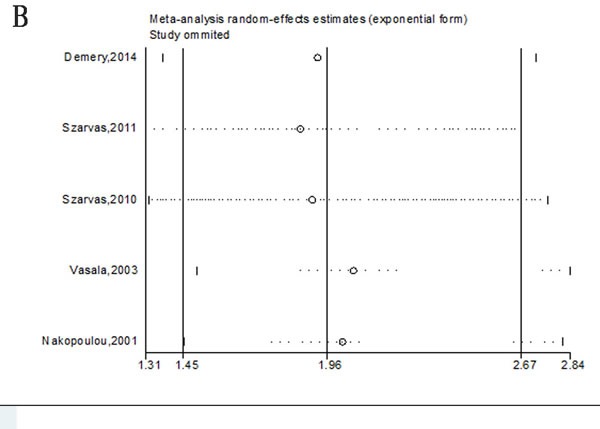

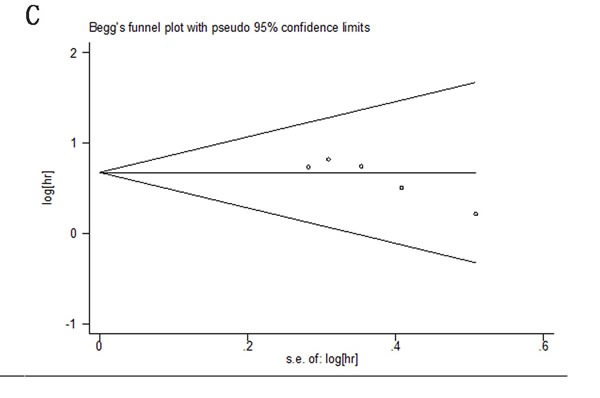
Meta-analysis of OS following exclusion of data from Vasala et al. (2008) **A**. Forest plots analysis of OS; **B**. Sensitivity analysis to confirmation of results’ stability; **C**. Publication bias to the evaluation of studies’ symmetry.

### Publication bias

In this meta-analysis, publication bias was evaluated using Begg's funnel plots and the Egger test (Figure 8). For the pooled analyses of OS, DSS, DFS/RFS, and PFS/MFS, *P* values of the Egger test were 0.076, 0.728, 0.887, and 0.725, respectively. Adding to this, the funnel plots were symmetrical and no obvious publication bias was identified.

## DISCUSSION

Ranking as the ninth most common carcinoma in 2008, bladder cancer is the most frequent neoplasm of the urinary tract worldwide [18, 19]. Although remarkable advance in treatment has been made recently, high mortality, recurrence rates, and poor prognosis are still the major concerns. Even after early radical cystectomy and reasonable drug therapy, quite a few patients with bladder carcinomas experience primary invasion and metastasis [20]. Transitional cell tumors account for 95% of bladder carcinoma types. The majority of patients develop recurrences after surgery and tend to progress to an advanced stage. Despite various combined therapy approaches, bladder carcinoma remains progressive with high relapse rates worldwide [21–22]. Therefore, investigating factors that are associated with tumor infiltration and metastasis may provide some appropriate therapies for different cases and judge their prognosis. Over the years, numerous potential biomarkers have been tested for predicting outcomes of bladder cancer patients.

Extracellular matrix (ECM) can provide structural support for cells and develop tissue frameworks that get involved in a dynamic process of interacting with cells and regulating their functions. Recent studies focusing on the mechanism of metastatic tumor dissemination have verified that ECM plays a key role in the multistep process of invasion and metastasis [23, 24]. In this process, tumor cells must secrete proteases or enhance relevant protease activities and possess the ability to degrade the ECM of the basement membrane and the intercellular matrix. The interaction between tumor and ECM is a prerequisite for tumor growth, and plays an important role in both the prognosis and progression of bladder cancer [25, 26].

The proteases secreted by tumor cells leading to the degradation of ECM are MMPs. Currently, MMPs family consists of 25 extracellular zinc endopeptidases, and they are the most important members of the proteases family [27]. MMPs family contains various subgroups such as: collagenases, gelatinases, stromelysins, matrilysins, membrane-type MMPs, and other subtypes. Among all kinds of the proteases listed above, MMP1, MMP8, and MMP13 are collagenases; MMP2 and MMP9 are gelatinases; MMP3, MMP10, MMP11, and MMP12 are stromelysins; while MMP7, MMP16, MMP14, MMP15, and MMP-26 are matrilysins, membrane-type MMPs, and other subtypes [28]. Previous studies have proven that MMPs can eliminate most of the ECM components, such as native collagen types, gelatin, fibronectin, and others [27]. In addition, MMPs have been considered as essential elements for invasive tumor growth and metastasis of bladder carcinoma [29]. In accordance with this, abnormal expression of MMPs was identified as a prognostic indicator in patients with bladder carcinoma [30, 31]. However, up to date most of clinical investigations focused on partial populations and specimens, which are unable to reveal the value of MMPs in survival outcomes of bladder cancer. Thus, we conducted this systematic review and meta-analysis to clarify the status of MMPs in bladder cancer populations. Although some review articles have investigated MMPs polymorphisms and their clinical association with the risk of bladder carcinoma, this is the first systematic review and meta-analysis evaluating the association between abnormal MMPs expression and patients’ outcomes [16, 30].

In this meta-analysis, we used subgroup, sensitivity, and heterogeneity analysis to explore the effects of main characteristics in the included studies. Our results indicated that overexpression of MMPs is a prognostic factor predicting poor bladder cancer survival. A pooled HR of 1.67 and 95% CI (1.26–2.22) in the OS analysis demonstrated that increased MMPs expression correlates with poor outcome in bladder cancer.

In contrast to OS, DSS can more accurately reflect the cause specific mortality of cancer, by excluding those that died from non-tumorous causes, such as cardiovascular, cerebrovascular, or others. According to the study of Gschwend et al. on bladder cancer patients undergoing radical cystectomy, the outcome is best characterized by DSS rather than OS, and they defined DSS as a better predictor for survival analyses [19, 32]. Our calculation of DSS/CSS analyses revealed a pooled HR of 1.66 (95%CI= 1.38–2.01), which demonstrated that high MMPs expression was associated closely with higher risk of disease-specific death risk (*P* = 0.041, *I*2 = 39.4%). In stratified analyses, we found that multiple MMPs tended to be prognostic for a poor bladder cancer-specific survival, suggesting the specific relationship between MMPs and survival rates. Furthermore, subgroup analyses based on assay methods, sample types, and ethnicity provided a quite unified conclusion that up-regulated MMPs predict inferior DSS. However, we could not ignore the individual study influence on the overall results. Among all the included studies, Svatek et al. in 2010 detected plasma MMP concentrations (MMP1, 2, 3, 7, 8, 9, and 12) in 135 bladder cancer patients, reporting that high MMP7 levels were significantly associated with poor CSS rates (HR= 2.24, 95%CI= 1.12–4.47; *P* = 0.022). Other MMPs did not show a statistically significant association (*P*> 0.05). In stratified analysis by MMPs type, the prognostic function of different MMPs was still controversial and unstable. We attributed this to the different molecular structures, limited relevant research, and therefore the stability of the conclusion needs to be further verified.

Furthermore, DFS/RFS analysis suggested that high MMPs expression did not develop a pivotal role in recurrence of bladder cancer (HR= 1.21, 95%CI= 0.96-1.53). However, subgroup analysis of pathology stage indicated that MMPs overexpression enhanced high relapse risk in patients with NMIBC but not MIBC. NMIBC patients treated with transurethral resection might account for the high recurrence rates, in contrast to MIBC patients undergoing radical surgery [21]. Other stratified analysis failed to achieve statistical significance. Subsequently, five studies focusing on PFS/MFS analysis showed that increased MMPs expression indicated high risk of tumor progression and metastasis (HR= 3.03, 95% CI= 1.98–4.64).

These significant findings implied the predictive role of MMPs in poor outcome of bladder carcinomas [33–35]. The majority of current investigations have provided strong evidence for the oncogenic role of MMPs in human bladder cancer. However, due to the limited literature quantities included in this meta-analysis, additional high-quality studies are still needed to further verify the conclusion.

Besides, there are seven included studies reported the association between MMP2 and bladder cancer, and six investigations focused on MMP9. Interesting, MMP2, together with MMP9, which belong to the gelatinases family, can degrade a major component of basement membrane named type IV collagen [9, 36, 37]. Contradictory results have been reported in previous studies about the prognostic value of MMP2 in bladder cancer. Vasala et al. and Kanayama et al. reported up-regulated MMP2 expression as a significant risk factor for poor DSS in patients with bladder carcinoma [38, 39]. However, Svatek et al. detected the concentration of MMP2 by ELISA, and found that MMP2 levels were unrelated to prognosis. These contradictory studies highlight the urgent necessity for the standardization of prognostic marker analysis in order to provide more convincing conclusion. Similar to MMP2, the prognostic role of MMP9 is equally disputed. In this meta-analysis, we found that most of articles regarded MMP9 as a prognostic factor for poor outcome while Vasala et al. and Svatek et al. found no correlation between MMP9 and outcomes [17, 40]. Thus, the relevance of MMP9 overexpression is highly questionable and still needs further confirmation. Our DFS/RFS analyses showed that only MMP2 and MMP9 were related to disease recurrence in patients. In consistent with four studies performing DFS/RFS analysis, Olsson et al. reported that abnormal expression of MMP2 and MMP9 were associated with a high risk of tumor recurrence in patients with stage T1 bladder cancer [41]. Vasala et al. and Hara et al. found that high MMP2 and MMP9 expression correlated with poor survival and high recurrence rate. In contrast, other two included articles reported that MMP2 and MMP9 was related to a low risk of recurrence [17, 42–43]. As previously explained, limitations of research quantities and neoplasm stages might account for these discrepancies. Besides, none of other MMPs in this meta-analysis were found to be associated with tumor recurrence. Mechanism and role of other MMPs involved in bladder carcinoma's recurrence is still not clear and requires further confirmation.

To conclude, our results indicated that detection of abnormal MMP levels is of great value in prognosis of bladder carcinoma patients. Although extensive retrieval was conducted in analysis, along with rigorous statistical analysis, our conclusion still needs cautious interpretation for several reasons. First, on account of limitation of articles reporting OS/DFS/PFS in this meta-analysis, the reliability and tightness of pooled HRs is not fully adequate. Second, several different assays such as IHC, ELISA, RT-qPCR, and northern blot were used to detect the concentration of MMPs in different samples. To some extent, methodological differences among individual investigations may contribute to inevitable heterogeneity. Additionally, even most of current studies have maintained a median or mean value as the cut-off point, a recognized MMPs expression level to define a single value was still difficult to achieve. Third, no independent investigation on Negroid was included in this meta-analysis, which might undermine the comprehensiveness to some extent. Furthermore, no prospective studies were available for this meta-analysis, and it might weaken the validity of results. Taking these limitations into account, the prognostic value of MMPs in bladder neoplasm might be overestimated. Our results should be interpreted rigorously because of these imperfections.

In conclusion, our analysis indicated that high MMPs expression significantly predicted poor OS/DSS/PFS in bladder cancer populations. However, overexpression of MMPs did not function as key factiors in bladde cancer relapse. Besides, only MMP2 and MMP9 were found to involve into cancer. Given the current insufficient evidence, further high quality investigations and large-scale studies are required to comfirm our findings, which can also develop more clinical applications and provide accurate prognostic information.

## MATERIALS AND METHODS

### Search strategy

To evaluate the specific role of MMPs in bladder carcinomas, we used several online databases including PubMed, EMBASE and the Web of Science to search relevant literature published through June 2016. Primarily, only studies published in English could be included to this meta-analysis. For the literature retrieval, following medical subject and text words were used: “bladder cancer” or “bladder carcinoma” or “bladder Neoplasm” or “bladder Tumor”, “Membrane-Type Matrix Metalloproteinase” or “MMPs” or “Matrix metalloproteinase” and “prognostic” or “prognosis” or “survival” or “outcome” or “recurrence” or “relapse”. In addition, following criteria should be considered to select the literatures: (1) an emphasis to human beings, (2) a relationship between MMPs and prognosis or survivals of bladder cancer. We also searched for Chinese articles to better understanding the association between MMPs and bladder neoplasm. Finally, no Chinese studies were meeting the inclusion criteria. Afterwards, articles published was retrieved for further checking. Additionally, we also screened the references of retrieved articles for any possible eligible studies.

### Quality assessment

MMPs are a family including multiple MMP types, such as: MMP1, MMP2, MMP7, MMP9, MMP11, MMP14 and so forth. The aim of this meta-analysis is to analyses the value of MMPs family to prognosis of patients with bladder carcinoma. Therefore, studies were selected only the patients were undergoing follow-up intervention and in whom expression levels of MMPs were measured. To evaluate the retrieval studies, articles published must be an original clinical study, for instance a case-control study or a randomized controlled trial. In addition, we also recorded following information: (1) the study population and country, (2) the type of MMPs, (3) dominant assay method to determine MMPs: ELISA, IHC or RT-qPCR, etc (4) the prognosis or survival assessment, (5) the detected sample and cut-off point of MMPs, (6) the follow-up period of patients, et al (Table 1). Sensitivity analyses and published bias were performed to promote the quality of this meta-analysis.

### Data selection

All data from eligible studies were extracted independently, ambiguous data were reviewed in detail. Extracted data elements comprised as follows: (1)the first author's name and publishing year; (2) the MMPs type of study, (3) the population, nationality, dominant ethnicity and detected sample; (3) the investigating method, cut-off value and follow-up time; (4) Hazard ratio associated with overexpression MMPs for overall survival (OS), disease-specific survival (DSS), disease-free survival (DFS)/recurrence-free survival (RFS) and progression-free survival (PFS)/metastasis-free survival (MFS) along with their 95% CI and P values, (5) the median or mean age of the patient. If HR and 95% CIs were not provided in studies, we extracted them from graphical survival curves using Engauge Digitizer version 4.1 [44, 45]. All relevant data mentioned above were comprehensively shown in Table 1 and Table 2.

### Statistical analysis

According to the heterogeneity of pooled studies, we choose the fixed-effects model (Mantel-Haenszel method) or the random-effects model (DerSimonian-Laird method) to analyse the data above. Heterogeneity test for pooled HRs was calculated by Cochran Q-test and Higgins I-squared statistic (*I*2). A random-effects model (DerSimonian-Laird method) was applied if *P*<0.10 or *I*2>50%, otherwise a fixed-effects model (Mantel-Haenszel method) was used instead [46]. Additionally, subgroup analysis based on similar factors were performed to reduce the influence of heterogeneity. We used Begg's funnel plot and Egger linear regression test with a funnel plot to evaluate the publication bias of eligible literature [47]. All statistical analyses were conducted with Stata12 (StataCorp LP, College Station, TX, USA), Review Manager (RevMan) V.5.3 (Copenhagen: the NordicCochrane Centre, the Cochrane Collaboration, 2014), Microsoft Excel (V.2013, Microsoft Corporation, Redmond, Washington, USA).
